# A new simple score of chronic cough: cough evaluation test

**DOI:** 10.1186/s12890-020-1106-1

**Published:** 2020-03-20

**Authors:** Wenzhi Zhan, Liting Zhang, Mei Jiang, Meihua Chen, Xiaoling Yuan, Jianxin Sun, Pusheng Xu, Feng Wu, Chunlai Zhang, Wei Luo, Xiaomei Chen, Hu Li, Kefang Lai

**Affiliations:** 1 State Key Laboratory of Respiratory Disease, National Clinical Research Center for Respiratory Disease, Guangzhou Institute of Respiratory Health, the First Affiliated Hospital of Guangzhou Medical University, 151 Yanjiang Rd, Guangzhou, Guangdong 510120 P. R. China; 2 Respiratory Department of the Third People’s Hospital of Dongguan city, Dongguan, Guangdong P. R. China; 3 Zhongshan Hospital of Sun Yat-sen University, Zhongshan People’s Hospital, Zhongshan, Guangdong P. R. China; 4 The Second People’s Hospital of Zhaoqing, Zhaoqing, Guangdong P. R. China; 5 Department of Respiratory, The Second Affiliated Hospital of Guangzhou Medical University, Guangzhou, Guangdong P. R. China; 6 Huizhou Third People’s Hospital, Guangzhou Medical University, Huizhou, Guangdong P. R. China; 7 Jiangmen Central Hospital, Jiangmen, Guangdong P. R. China

**Keywords:** Chronic cough, Severity, Quality of life, Evaluation test, Validation

## Abstract

**Background:**

Chronic cough has an important impact on physical, social and psychological aspects. A simple and effective method to assess different aspects of chronic cough severity is required. We aimed to develop a simple, self-completed test, Cough Evaluation Test (CET), to evaluate cough severity and its impact on health.

**Method:**

The items of preliminary CET were generated based on literature review and clinical practice. Items reduction was conducted by modified Delphi method. Patients with chronic cough were recruited to complete CET, Cough Visual Analog Scales (VAS), Mandarin Chinese version of the Leicester Cough Questionnaire (LCQ-MC), and Cough Symptom Score (CSS). Reassessments were performed at 1 week apart before treatment, and after more than 2 weeks treatments. Concurrent validation, internal consistency, repeatability, responsiveness and the minimal important difference (MID) were determined.

**Results:**

CET consists of five items with a 5-point Likert scale (1–5 scaling of items, 5–25 score range). The Cronbach’s alpha values for CET was 0.80. CET showed a stronger correlation with LCQ-MC (*r* = − 0.74) compared to that between LCQ-MC with VAS (*r* = − 0.61). CET also showed a stronger correlation with VAS (*r* = 0.70) compared to that between VAS with other measures. Intraclass correlation coefficients for CET was 0.84. In patients undergoing treatment, CET scores significantly changed (*p* < 0.0001). The MID of CET was 2.

**Conclusion:**

Cough Evaluate Test is a reliable, valid and responsive tool to simply evaluate impact of cough on physical, social and psychological aspects.

## Background

Chronic cough was a common complaint of patients in respiratory clinics, which was involved in many conditions, such as cough variant asthma, eosinophilic bronchitis, atopic cough, upper airway cough syndrome and gastro-oesophageal acid reflux disease [1, 2]. The global prevalence of chronic cough was up to 9.6% in general adult populations [3]. Chronic cough could cause significant physical, psychological and social morbidity. Besides routine clinical evaluations, reliable and valid evaluation on the impact of cough on their health status is an important step in management of the patients. An ideal measure for chronic cough should include information on cough severity, social and psychological aspects of patients and promote communication between patient and clinician. A few measurements have been used in clinical practice and clinical trials. Cough Symptom Score (CSS) was a simple two-part questionnaire relating to cough symptoms [4], which is focused on cough frequency, and overall impact on daily life. However, CSS is not easy to distinguish the different score level based on cough numbers, and there was little clinical experience with this tool. Cough Visual Analog Scales (VAS) is a brief subjective assessment of cough severity, but lost sight of other aspects of cough-impact on health. Available cough-specific health status measures, such as the Leicester Cough Questionnaire (LCQ) [5], and Cough-Specific Quality of-Life Questionnaire (CQLQ) [6], were reliable, valid, widely used in clinical trials. It takes more than 5 min to complete and calculate score of CQLQ with 28 items in six domains, and it needs scoring algorithms to calculate scores of each domain and total score of LCQ with 19 items in three domains, which makes these questionnaires are time-consuming and complex to use in clinical practice. There is a lack of a simple, effective method to assess different aspects of chronic cough in routine care [7, 8]. Therefore, we aim to develop a new simple measurement for chronic cough, Cough Evaluation Test (CET), including physical, psychological and social aspects.

## Method

### Item generation

Preliminary CET were first generated in reference to LCQ and clinical practice with the following criteria: 1) including three aspects of physical, psychologic, and social impact;2) integrating or subtracting similar or redundant items; 3) adding some items which are important in chronic cough management. The preliminary list of items was distributed to a Delphi expert panel. We invited 57 respiratory experts who are the fellow members of Chinese Thorax Society and specialists on field of chronic cough to evaluate the importance of items in the CET. Two Delphi rounds were conducted for comment on the list and identify further indicators. For each Delphi round, electronic surveys were used to collect the data. The Delphi group members were asked to rate the importance of each item in preliminary CET, which has 13 items on a 5-point scale (1 = not important, 5 = extremely important). If the expert disagrees with the formulation of the item, explanation should be provided. For the second Delphi round, experts were asked to rate each item again, and they could change their score in view of the group’s response to the previous round. Electronic reminders were sent to non-responders 1 and 2 weeks later.

Response scale: A 5-point Likert scale was used to rate the items of CET ranging from 1 (none of the time) to 5 (all of the time.) A higher score indicated worse condition of cough. The overall score for the CET was calculated by adding the scores of each item.

### Validation

#### Subjects

Patients with chronic cough (> 14 years of age) were recruited from the respiratory outpatient clinic. Chronic cough was defined as cough being sole or predominant symptom lasting more than 8 weeks, without overt identifiable abnormalities on chest X-ray. The study was approved by the Ethics Committee of the First Affiliated Hospital of Guangzhou Medical University. (Number:201777).

#### Reliability and item analysis

Internal reliability was assessed by determining Cronbach’s alpha coefficients which indicated the extent to which items are related. Internal reliability is generally acceptable if Cronbach’s alpha coefficient is greater than 0.7. Inter-item correlations were assessed for further evidence of homogeneity and potential redundancy of items. For example, when the inter-item correlation was greater than 0.7, it indicated that the items were similar. Items were also omitted when the item to total score correlation was less than 0.2, indicating little contribution to the overall score. Items with ceiling or floor effect (> 50% of the respondents chose an extreme positive or negative response category, respectively) would be removed.

#### Concurrent validity

Concurrent validity was assessed by correlating scores of CET with three health outcome measures completed at the same time: (1) VAS is a 100 mm scale on which patients indicate the severity of cough. (2) The Leicester Cough Questionnaire (LCQ) is a 19-item questionnaire that assesses cough related quality of life [5]. LCQ-MC was used and presented as its total score in this study, which had already been validated in bronchiectasis and in non-small cell lung cancer patients after surgery [9, 10]. (3) CSS is a two-part questionnaire relating to cough symptoms during the day and at night [4].

#### Repeatability

The test-retest procedure measured the stability of scores on CET over time in patients who had a stable condition of chronic cough. The repeatability of the CET was assessed in those patients indicating no change in cough status in 1 week apart. LCQ-MC, VAS and CSS were also completed by the subjects in the same time.

#### Responsiveness

Responsiveness of CET, LCQ-MC, VAS and CSS were tested before and after at least 2 weeks treatment. Individual treatment for chronic cough was based on its etiology according to the principle of cough guideline [11]. Patients were asked if their cough had improved after treatment.

#### Statistical analysis

SPSS version 16 was used for data analysis. Data are presented as the mean (standard deviation) or median (range) according to its distribution. Statistical comparisons among groups were performed with one-way analysis of variance (ANOVA) for normally distributed data and Kruskal–Wallis tests for skewed data. Spearman’s correlation coefficient was used to determine concurrent validity. Comparison of the magnitude of two correlations was referred to the method described previously [12]. Internal consistency and reliability were evaluated by calculating Cronbach’s alpha coefficients. Repeatability testing were assessed using intraclass correlation coefficients and Bland-Altman plot. Responsiveness was analyzed by paired t-test and effect size for these measures scores that was determined by the difference in mean total the measure score before and after the treatment/SD of the measure score before the treatment. The Minimal Important Difference (MID) of CET was assessed with distribution-based method [13, 14] in which the values were calculated from the standard error of measurement (SEM). SEM = SD √(1-R). SD means standard deviation at baseline, and R means Intraclass correlation coefficients.

## Results

### CET generation

There were 13 items in the preliminary CET regarding physiology, social and psychological aspects. Items with physiologic aspect included cough frequency of different time (day or night), intense cough, cough beyond control, cough disturbed sleep, sputum (phlegm) production and difficulty to expectorate (Table 1). Items in regard of social aspect was presented as how cough interfered daily life, which consist of three aspects: whether cough interfered with working, daily activities, colleagues or families. Items related with psychological aspect included psychological burden, embarrassment, feeling anxious or depressive and concerning whether cough was related to infectious conditions or lung cancer. Among 57 respiratory experts who were invited to evaluate the importance of items in the CET, 33 experts responded and participated in round 1 evaluation. In the second Delphi round, experts who participated in previous round were asked to rate the item again. The response rate for round 2 was 93.94% (31/33). The mean ratings and the percentage of item-rated-score at least 4 were presented in Table 1. The top 5 items were “cough during the day”**,** “cough during the night”, “cough disturbed sleep”, “cough interfered with daily life”, and “intense cough”. Considering the objective to evaluate of “cough disturbed sleep?” and “cough during the night” are similar, hence these two items were merged into one item. As items of CET were supposed to contain physical, psychological and social aspects of chronic cough, “feel anxious or depressive” were selected to enter the final version of CET (Table 2).

**Table 1:** Results for Delphi round 1 and 2

	Round 1		Round 2	
	Mean ± SD	Scores≥4, %	Mean ± SD	Scores≥4, %
**1. Cough frequency during the day**	4.35 ± 0.55	96.77%	4.44 ± 0.50	100.00%
2. Cough frequency during the night	4.58 ± 0.56	96.77%	4.72 ± 0.46	100.00%
3. Cough frequency during the day and night	4.00 ± 1.12	75.00%	4.10 ± 0.98	82.76%
**4. Intense cough**	4.07 ± 0.70	79.31%	4.17 ± 0.65	86.67%
5. Cough beyond control	3.79 ± 1.15	62.07%	4.10 ± 1.06	70.00%
**6. Cough disturbed sleep**	4.58 ± 0.62	93.55%	4.77 ± 0.50	96.77%
**7. Cough interfered with daily life**	4.40 ± 0.72	86.67%	4.66 ± 0.55	96.88%
8. Psychological burden from cough	3.52 ± 0.94	48.15%	3.54 ± 0.79	53.57%
**9. Feel anxious or depressive**	3.80 ± 0.89	63.33%	3.87 ± 0.72	74.19%
10. Feel embarrassment	3.21 ± 0.86	34.48%	3.27 ± 0.64	36.67%
11. Concerned that cough was related to infectious disease or lung cancer	2.88 ± 1.21	30.77%	2.90 ± 1.01	27.59%
12. Sputum (phlegm) production	4.58 ± 0.72	93.55%	3.81 ± 0.82	68.75%
13. Difficult to expectorate	3.84 ± 0.82	77.42%	3.94 ± 0.72	84.38%

**Table 2:** Cough Evaluation Test

Please read each question carefully to assess your condition at present and answer by ‘√’ the response that best applies to you.	None 1	Seldom 2	Sometimes 3	Often 4	All of the time 5
How frequently did you cough during the day?	1	2	3	4	5
Have your cough disturbed your sleep?	1	2	3	4	5
Did you have intense cough?	1	2	3	4	5
Have your cough interfered with your daily life?	1	2	3	4	5
Have your cough made you feel anxious or depressive?	1	2	3	4	5
			Total score:		

### Participant characteristics

We recruited 279 chronic cough patients. Forty patients were excluded due to cough duration less than 2 months (15), poor education (7), or omission of some items of questionnaires (18). The characteristics of the remaining 237 patients were showed in Table 3. The mean age of patients was 40.4 ± 12.5 years, and female accounted for 128 (54%). The scores for LCQ-MC, cough VAS, CET and CSS were also listed. After the first assessment, fifty-nine patients were recruited to complete CET and other measures in 1 week apart before the treatment. And we randomly recruited 100 patients who had been assessed before and after more than 2 weeks treatment for responsiveness analysis. The causes of these 100 patients were gastro-oesophageal reflux (*n* = 24), cough variant asthma (*n* = 20), upper airway cough syndrome (*n* = 12), atopic cough (*n* = 8), chronic bronchitis (*n* = 7), bronchiectasis (*n* = 5), and other conditions (*n* = 24).

**Table 3:** Baseline characteristics of the study population

	All participants	Repeatability	Responsiveness	*p* value
Cases	237	59	100	
Age, years	40.4 ± 12.5	37.4 ± 11.2	40.4 ± 12.6	0.2286
Gender (% female)	54%	46%	57%	0.5054
Duration of cough, months	43.0 ± 77.3	39.1 ± 62.1	36.1 ± 66.5	0.7415
LCQ-MC	12.9 ± 3.6	13.6 ± 3.8	13.1 ± 3.7	0.4777
VAS, mm	58.9 ± 21.0	52.6 ± 20.2	54.4 ± 20.8	0.2942
CET	13.9 ± 4.2	12.8 ± 4.1	14.0 ± 4.1	0.1534
CSS				
Daytime	2.9 ± 0.8	2.7 ± 0.8	2.9 ± 0.8	0.1213
Nighttime	1.3 ± 1.0	1.0 ± 1.0	1.4 ± 1.1	0.0685
Total	4.3 ± 1.5	3.7 ± 1.5	4.3 ± 1.4	0.0235

Data were presented as Mean ± SD. *LCQ-MC* Mandarin Chinese version of the Leicester Cough Questionnaire, *VAS* Visual Analog Scales, *CET* Cough Evaluation Test, *CSS* Cough Symptom Score

### Internal consistency and item analysis

The Cronbach’s alpha value of CET was 0.80. No item had a ceiling or a floor effect. Inter-item correlations were mild to moderate (Table 4). No items were removed from this analysis as no items had consistently high or low correlations. All items had corrected item-total correlation coefficients greater than 0.4 and less than 0.7 (Table 5). Cronbach’s alpha was high for all items, and there was no substantial increase with removal of these items, thus all items were retained.

**Table 4:** Inter-Item Correlation Matrix of the CET (*N* = 237)

	Cough during the day	Cough disturbed sleep	Intense cough	Cough interfered with daily life	Feel anxious or depressive
Cough during the day	1	0.27	0.47	0.43	0.33
Cough disturbed sleep	0.27	1	0.52	0.46	0.44
Intense cough	0.47	0.52	1	0.56	0.47
Cough interfered with daily life	0.43	0.46	0.56	1	0.62
Feel anxious or depressive	0.33	0.44	0.47	0.62	1

**Table 5:** Item reliability for the 5 items of CET (*N* = 237)

	Scale Mean if Item Deleted	Scale Variance if Item Deleted	Corrected Item-Total Correlation	Squared Multiple Correlation	Cronbach’s Alpha if Item Deleted
Cough during the day	10.35	13.98	0.47	0.26	0.80
Cough disturbed sleep	11.73	11.64	0.55	0.33	0.79
Intense cough	11.22	11.56	0.66	0.45	0.75
Cough interfered with daily life	11.32	10.56	0.69	0.50	0.74
Feel anxious or depressive	11.13	11.28	0.62	0.42	0.76

### Concurrent validity

CET showed strong correlations with VAS, LCQ-MC and CSS total scores respectively (*r* = 0.70, *r* = − 0.74, and *r* = 0.71, respectively). There were just mild to moderate correlation between CSS and LCQ- MC, CSS and VAS at baseline (Table 6). And the correlation between cough VAS and LCQ-MC at baseline was − 0.61 (*P* < 0.0001). The strength of correlation between CET and VAS was significantly stronger than that between VAS and LCQ-MC /CSS daytime /CSS nighttime at baseline. And the strength of correlation between CET and LCQ-MS was significantly stronger than that between LCQ-MS and other measures at baseline.

**Table 6:** Spearman rank correlations between different health outcome measures at baseline (*N* = 237)

	CET	VAS	LCQ-MC	CSS Total	CSS daytime	CSS nighttime
CET		0.70^*#^	−0.74^*&^	0.71*	0.60*	0.53*
VAS	0.70^*#^		− 0.61*	0.66*	0.60*	0.43*
LCQ-MC	−0.74^*&^	− 0.61*		− 0.54*	− 0.50*	− 0.38*
CSS Total	0.71*	0.66*	−0.54*		0.76*	0.79*
CSS daytime	0.60*	0.60*	−0.50*	0.76*		0.23*
CSS nighttime	0.53*	0.43*	−0.38*	0.79*	0.23*	

* *P* < 0.0001

# correlation between CET and cough VAS versus correlation between VAS and LCQ-MS or correlation between VAS and CSS daytime/nighttime (*P* < 0.0001, *P* = 0.0143, *P* < 0.0001, respectively)

& correlation between CET and LCQ-MC versus correlation between VAS and LCQ-MS or correlation between LCQ-MS and CSS total/daytime/nighttime (*P* = 0.0002, *P* < 0.0001, *P* < 0.0001, *P* < 0.0001, respectively)

There were much better correlations between CET and other outcome measures after 2 weeks treatment, compared to that at baseline (Table 7). And the correlation between VAS and LCQ-MC was − 0.76 after 2 weeks treatment. Moreover, after 2 weeks treatment, the strength of correlation between CET and LCQ-MC was significantly higher than that between LCQ-MS and other measures as well. In addition, the correlation between CET and VAS was significantly stronger than correlations between VAS and LCQ-MC /CSS total /CSS nighttime.

**Table 7:** Spearman rank correlations between different health outcome measures after treatment (*N* = 100)

	CET	VAS	LCQ-MC	CSS Total	CSS daytime	CSS nighttime
CET		0.87*^#^	−0.85*^&^	0.75*	0.78*	0.49*
VAS	0.87*^#^		−0.76	0.78*	0.82*	0.45*
LCQ-MC	−0.85*^&^	−0.76*		−0.76*	− 0.72*	−0.56*
CSS Total	0.75*	0.78*	−0.76*		0.84*	0.78*
CSS daytime	0.78*	0.82*	−0.72*	0.84*		0.37*
CSS nighttime	0.49*	0.45*	−0.56*	0.78*	0.37*	

* *P* < 0.0001

# correlation between CET and VAS versus correlation between VAS and LCQ-MS or correlation between VAS and CSS total/nighttime (*P* < 0.0001, *P* = 0.0082, *P* < 0.0001, respectively)

& correlation between CET and LCQ-MC versus correlation between VAS and LCQ-MS or correlation between LCQ-MS and CSS total/daytime/nighttime (*P* = 0.0015, *P* = 0.0145, *P* = 0.0004, *P* < 0.0001, respectively)

### Repeatability testing and the MID

**Fig. 1:**
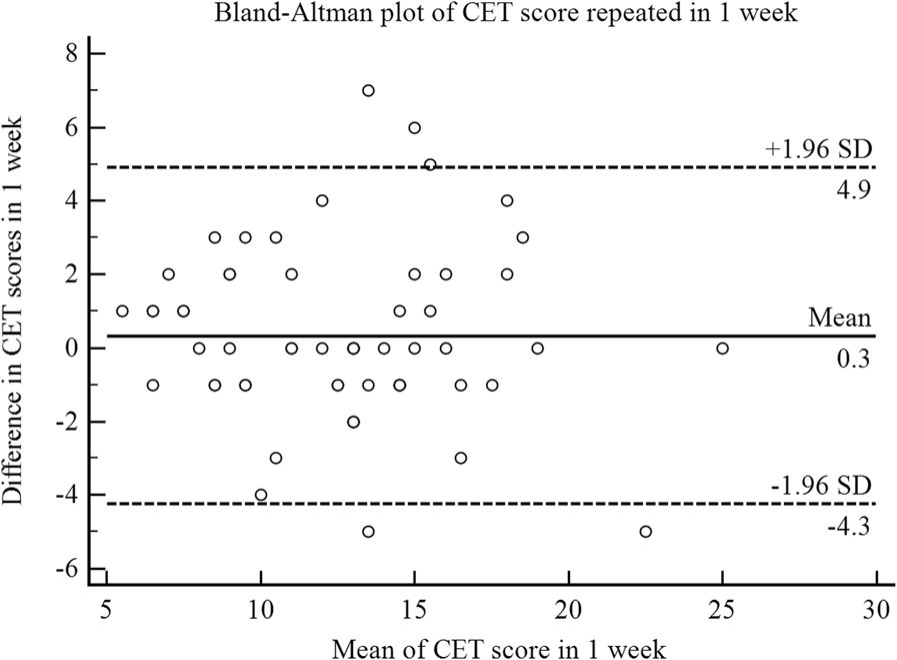
Bland-Altman plot of CET score repeated in 1week in 59 patients with chronic cough. ––– = mean difference between the two scores; - - - - = 95% limits of agreement

Intraclass correlation coefficients for CET, LCQ-MC, and VAS were high (0.84, 0.85, 0.85 respectively), while for the CSS were modest (daytime 0.64, nighttime 0.65, and total score 0.72). A Bland-Altman plot of CET score was shown in Fig. 1. The MID of CET was 2.

### Responsiveness testing

Following treatment based on cough treatment guideline [11], CET, VAS, LCQ-MC and CSS scores changed (*p* < 0.0001, Table 8). The effect sizes for change of these measures were presented in Table 8. There were strong relationship between change in CET score and that in VAS score (*r* = 0.72, *p* < 0.0001), LCQ scores (*r* = − 0.70, *P* < 0.0001), and CSS total scores (*r* = 0.66, *P* < 0.0001).

**Table 8:** Comparison of pre-post treatment CET, LCQ-MC, VAS and CSS scores in the responsiveness component of the study (*n* = 100)

	CET	LCQ-MC	VAS	CSS total	CSS daytime	CSS nighttime
Pre-treatment	14.0 (4.1)	13.1 (3.7)	54.4 (20.8)	4.3 (1.4)	2.9 (0.8)	1.4 (1.1)
Post-treatment	10.2 (3.9)	15.6 (3.7)	32.2 (22.0)	2.8 (1.6)	1.9 (1.2)	0.9 (0.9)
*P* value for paired t-test	*P* < 0.0001	*P* < 0.0001	*P* < 0.0001	*P* < 0.0001	*P* < 0.0001	*P* < 0.0001
Effect sizes	0.9	0.7	1.1	1.1	1.2	0.5

Data are presented as mean (SD)

## Discussion

This study has created a short, simple patient-completed test for chronic cough patients with good measurement properties. Almost all patients could complete the CET within 1 minute in this study. The 5 items selected for CET parallel the dimensions of cough management—cough severity, social impact and psychological effect. In this study, scores computed from CET were shown to be highly repeatable and responsive, suggesting CET may be a useful outcome measure in assessing the response to intervention in clinical practices and trials.

Cronbach’s alpha coefficient of CET was 0.80, which was sufficient in internal consistency. No matter at baseline or after treatment, CET has shown a strong correlation with cough VAS and LCQ-MC, and its correlation intensity were significantly higher than that between cough VAS and LCQ-MC, and were also higher than those between cough VAS or LCQ-MC and other measures, indicating CET was useful to assess cough severity and cough-related quality of life. Although intraclass correlation coefficient of LCQ-CM was lower than that in the previous researches (0.89–0.96) [5, 9, 10], but it still has excellent level of repeatability. The intraclass correlation coefficient of cough VAS (0.85) was almost the same as Birring’s data (0.84) [5], indicating VAS also has a stable and excellent test-retest reliability. In addition, CET has the same excellent degree of test-retest reliability as LCQ-MC and cough VSA did, and Bland-Altman plot of CET also showed its excellent repeatability.

Our data also showed that the CET was responsive to change after treatment and the effect size was less than that seen with the cough VAS, but was more than that seen with LCQ-MC. Cohen proposed benchmarks that serve to guide the interpretation of effect sizes: 0.2 for ‘small’ effects, 0.5 for ‘medium’ effects, and 0.8 for ‘large’ effects [15]. In our study, CET and cough VAS can detect “large” effects, while LCQ-MC can just reflect the “medium” effect after the treatment, suggesting that CET and cough VAS might be better outcome measures of choice in clinical trials. The effect size represents individual change in terms of the number of pre-test SD, which mean that characteristics of the distribution, particularly at baseline, may strongly influence the effect size [13]. And it could explain why effect sizes in our data were less than that in Birring’ result [5].

More than 80% of cough occurs during awake time [16], while some patients like cough variant asthma may also suffer from nocturnal cough. One direct effect of nocturnal cough was sleep disturbance. Moreover, cough intensity was also an important determinant of cough severity in some patients [16, 17]. Three items of CET including “How frequently did you cough during the day?”, “Have your cough disturbed your sleep?”, “Did you have intense cough?” correspond to cough severity that was a single concept with three inter-related components: frequency, intensity, and disruption [18]. Cough is protective reflex. Some smokers may cough a lot, but that may not be a big deal for them, while some patients who are social or have to give a speech frequently, coughing may be a huge issue. In addition to physical discomfort, a protracted cough could also cause anxiety, social and personal embarrassment, and deterioration in quality of life especially in psychosocial condition [19, 20], which was also reflected in the last two items of CET with “Have your cough interfered with your daily life?”, and “Have your cough made you feel anxious or depressive?” . The CET correlated well with cough VAS and LCQ-MC, suggesting its great capability of assessing cough severity and cough-specific quality of-life. However, for chronic cough patients, especially for those who were diagnosed as unexplained or refractory chronic cough whose pathogenesis is inadequately understood and effective treatment is still lacking, whether CET is appropriated tool for long-term management and which cutoff score of CET is appropriate to identify the need of drug therapy still require further research.

Koo, Hyeon-Kyoung et al. have described the validation of another cough assessment test (COAT) [21]. Unlike the full evaluation of CET on cough severity, social impact and psychological effect, the COAT focuses on cough frequency, limitation on daily activities, sleep disturbance, fatigue and hypersensitivity to irritants, no sight of cough intensity and psychological impact. Further work is necessary to compare the CET and COAT in the evaluation of chronic cough.

There are limitations to our study. The MID of CET was just based on distribution-based method. Several studies have showed that a MID based on anchor-based approach was close to the value of one SEM [22, 23], while a few discrepancies were showed in other researches [24, 25]. It seems that one SEM equals the MID is not a universal truth [26]. In addition, the subjects selected for the test validation were older than 14 years without overt identifiable abnormalities on chest X-ray, which may restrict its application in children and those with abnormal chest radiograph.

## Conclusion

We develop a new cough evaluation test consisting 5 items. Through assessment with concurrent validation, internal consistency, repeatability, responsiveness and the minimal important difference, CET is a reliable, valid and responsive tool to simply evaluate full impact of chronic cough in regard of physical, social and psychological aspects. CET would facilitate an easy and efficient way to assess chronic cough in routine care and clinical trials.

## Data Availability

The datasets used and/or analyzed during the current study are available from the corresponding author on reasonable request.

## Abbreviations

CET: Cough Evaluation Test
COAT: Cough assessment test
CSS: Cough Symptom Score
LCQ: Leicester Cough Questionnaire
LCQ-MC: Mandarin Chinese version of the Leicester Cough Questionnaire
MID: The minimal important difference
VAS: Visual analog scales
